# Associations Between Bioavailable Vitamin D and Remnant Cholesterol in Patients With Type 2 Diabetes Mellitus

**DOI:** 10.7759/cureus.13248

**Published:** 2021-02-09

**Authors:** Gulsum Feyza Turkes, Sezer Uysal, Tevfik Demir, Yucel Demiral, Baris Onder Pamuk, Husnu Yılmaz, Leyla Demir, Mehmet Doruk, Giray Bozkaya

**Affiliations:** 1 Biochemistry, Faculty of Medicine, Dokuz Eylul University, Izmir, TUR; 2 Biochemistry, Kecioren Training and Research Hospital, Ankara, TUR; 3 Endocrinology and Metabolism, Faculty of Medicine, Dokuz Eylul University, Izmir, TUR; 4 Public Health, Faculty of Medicine, Dokuz Eylul University, Izmir, TUR; 5 Endocrinology and Metabolism, Izmir Katip Celebi University Atatürk Training and Research Hospital, Izmir, TUR; 6 Biochemistry, Izmir Katip Celebi University Atatürk Training and Research Hospital, Izmir, TUR; 7 Endocrinology and Metabolism, Izmir Bozyaka Training and Research Hospital, Izmir, TUR; 8 Biochemistry, Izmir Bozyaka Training and Research Hospital, Izmir, TUR

**Keywords:** 25 (oh) vitamin d, vitamin-d deficiency, vitamin d status, vitamin d-binding protein, bioavailable vitamin d, lipoprotein cholesterol, remnant cholesterol, diabetes mellitus type 2

## Abstract

Introduction

In circulation, 99% vitamin D is transported by binding to vitamin D binding protein (VDBP) and albumin. Vitamin D at free form and vitamin D binding to albumin are defined as bioavailable vitamin D. Vitamin D deficiency is associated with atherogenic lipid profile and insulin resistance. Remnant cholesterol is defined as the cholesterol component of triglyceride-rich lipoproteins and contributes to the atherosclerotic burden. The aim of this study was to investigate the association between bioavailable vitamin D and remnant cholesterol in patients with type 2 diabetes mellitus (T2DM).

Methods

A total of 198 T2DM patients and 208 non-diabetic subjects underwent biochemical measurements of lipid profiles, 25(OH)D, VDBP, CRP and albumin levels. Their demographic characteristics (age, sex) were questioned. Subjects with thyroid, kidney and liver dysfunction and using lipid-lowering therapy were not included in the study. The diagnosis of T2DM was made according to the American Diabetes Association* *ADA 2016 criteria. Classification of vitamin D levels was done according to the Endocrine Society. Bioavailable vitamin D concentrations were calculated.

Results

High-density lipoprotein cholesterol (HDL), 25(OH)D, free vitamin D and bioavailable vitamin D levels were significantly lower in diabetic patients than in non-diabetic patients while triglyceride, remnant cholesterol and CRP levels were found to be significantly higher. VDBP was positively correlated with CRP and remnant cholesterol in diabetic patients, but not in non-diabetic patients. Cut-off values were determined from non-diabetics as 3.56 ng/mL for bioavailable vitamin D and 26.56 mg/dL for remnant cholesterol. Logistic regression analysis in the control group showed that the odds ratio for increasing remnant cholesterol above the cut-off value was determined as 2.01 for low bioavailable vitamin D and 1.1 for elevated CRP. However, in T2DM there was no significant relationship. In all subjects, low bioavailable vitamin D increased the remnant cholesterol above the cut-off by 2.18-fold independent of the presence of T2DM. However, there was no significant risk to increase remnant cholesterol, considering a total 25(OH) D deficiency in all groups.

Conclusions

Low bioavailable vitamin D was found to be a risk factor for elevated remnant cholesterol. This relationship was not detected in patients with T2DM. We believe that the inflammation observed in Diabetes Mellitus may increase the concentrations of VDBP and a decrease in bioavailable vitamin D levels. Therefore, measuring VDBP and calculating the bioavailable vitamin D may provide additional information about the actual vitamin D status.

## Introduction

Type 2 diabetes mellitus (T2DM) is a metabolic disease resulting from beta-cell dysfunction, insulin resistance or inability to suppress glucose production by the liver. Patients with T2DM have abnormal post-absorptive lipid metabolism. Postprandial clearance is delayed even in patients with normal fasting levels of most lipid parameters including triglyceride (TG) and chylomicrons [1]. Therefore, diabetic patients have high TG-rich lipoprotein ratios.

Remnant cholesterol is defined as the cholesterol component of TG-rich lipoproteins (fasting very low-density lipoprotein [VLDL] and intermediate-density lipoprotein [IDL], plus postprandial chylomicrons and their remnants) [2]. Epidemiological studies offer evidence that remnant lipoproteins infiltrate the intima and lead to atherosclerosis similar to low-density lipoprotein (LDL), due to their cholesterol component [3]. Therefore, it is speculated that high levels of remnant cholesterol may be a predictive marker of future cardiovascular disease. In addition, recent studies have shown that LDL-cholesterol (LDL-C) causes atherosclerosis without inflammation, whereas remnant cholesterol has been associated with low-grade inflammation and all-cause mortality [2,3].

Vitamin D deficiency has been observed with high plasma levels of remnant cholesterol and LDL-C, and low plasma levels of high-density lipoprotein cholesterol (HDL-C) [1]. Vitamin D deficiency has also been associated with a higher incidence of ischemic coronary disease and T2DM [4]. Circulating vitamin D exists partly as a bioavailable free or weakly albumin-bound form, and partly as an inactive form bound to vitamin D binding protein (VDBP) [5,6]. VDBP inhibits the effect of vitamin D on target cells. Individual differences in VDBP levels and therefore bioavailable vitamin D levels suggest a possible relationship between vitamin D and cardiovascular disease.

The aim of this study was to investigate the correlation between bioavailable vitamin D and remnant cholesterol in patients with T2DM. 

## Materials and methods

Study design and population

This research was designed as a cross-sectional study and was conducted between November 2015 and December 2017. The study initially included 471 volunteers between 18 and 70 years of age who presented for diagnosis or follow-up to the endocrinology outpatient clinics of three major hospitals (Dokuz Eylul University Medical Faculty Hospital, Izmir Katip Celebi University Ataturk Training and Research Hospital, and Izmir Bozyaka Training and Research Hospital) in Izmir in Turkey. Samples were taken from volunteers who did not receive any medical treatment affecting lipid metabolism (e.g., statins, fibrates, niacin, retinoic acid, cyclosporine) after fasting for 8-12 hours. After excluding patients with thyroid, kidney, and liver dysfunction, the study was conducted with a total of 406 individuals consisting of 208 non-diabetic (NDM) individuals and 198 T2DM patients.

The study is registered under clinical trial number ChiCTR-ROC-16008613. Ethical approval was obtained from Dokuz Eylul University Medical Faculty Non-invasive Clinical Research Ethics Committee prior to the study. Individuals willing to participate in the study were asked to read and sign an informed voluntary consent form. We classified 25(OH)D levels according to the Endocrine Society. In a preliminary study that the poster presentation in congress we classified 25(OH)D levels according to the Endocrine Society and determined that remnant cholesterol levels were significantly higher than adequate in the presence of vitamin D deficiency. OpenEpi software was used for sample size analysis based on a 95% confidence level and 80% power.

Biochemical assays

Glucose, cholesterol, HDL-C, LDL-C, TG, thyroid-stimulating hormone (TSH) and CRP levels were analyzed by routine laboratory methods at the time of sampling. Direct LDL-C measurement was done in patients with TG levels of 400 mg/dL or higher. The Friedewald formula was used for those with TG values below 400 mg/dL. Blood samples were centrifuged immediately and the serum was stored at −80°C for analysis.

25(OH)D was measured using high-performance liquid chromatography (HPLC) (Shimadzu, Japan). Serum VDBP concentrations were measured using enzyme-linked immunosorbent assay (Elabscience, Houston, TX). The intraassay coefficient of variation (CV) was 7.8% and the interassay CV was 18.7% in our laboratory. Free and bioavailable vitamin D concentrations were calculated according to the mathematical equations of Bikle et al, using measured values of 25(OH)D, VDBP, albumin and affinity constants [5,7]. Results were defined as vitamin D deficiency when their bioavailable vitamin D values were equal to or below the 5th percentile of the value distribution in the NDM group with sufficient vitamin D levels [8].

Remnant cholesterol was calculated by subtracting HDL and LDL cholesterol from total cholesterol. A remnant cholesterol threshold value was determined based on the 95th percentile value among subjects in NDM group with TG <150 mg/dL and total cholesterol <200 mg/dL. Values higher than this level were classified as high and those below this level were considered normal.

Statistical analyses

SPSS for Windows version 23.0 software (IBM Corp., Armonk, NY) was used for statistical analyses. Parameters were evaluated for normal distribution with the Kolmogorov-Smirnov test. In univariate analysis, the Mann-Whitney U test was used to compare the means of continuous data. Correlation analyses were done using Spearman’s test. Chi-square analyses were used for comparisons of discrete variables and the results were presented with continuity correction.

In the main analysis was done to test our hypothesis that low level of bioavailable vitamin D is a risk factor for remnant cholesterol, Logistic regression models were constructed. In logistic regression analysis, variables associated with remnant cholesterol in univariate analysis and potentially confounding variables were used to create models for NDM individuals, DM patients, and the whole group. In models 1 and 2, age, sex, CRP, and bioavailable vitamin D were used as independent variables in the NDM individuals and DM patients, respectively. In model 3, age, sex, CRP, bioavailable vitamin D, and T2DM were used as independent variables for the entire study group. The interaction of bioavailable vitamin D and T2DM for remnant cholesterol was analyzed by including the multiplicative variable “bioavailable vitamin D x T2DM”. The results were presented as odds ratio (OR) and 95% confidence interval (CI). p values less than 0.05 were considered statistically significant.

## Results

The study group included a total of 406 adults between the ages of 18 and 70 years. The patients in the T2DM group were significantly older than those in the NDM group (54 ± 9 vs. 45 ± 12 years, p<0.001). In terms of gender distribution, 79 (39.9%) of the T2DM group were men, compared to 47 men (22.6%) in the NDM group. The T2DM group had a significantly higher male to female ratio (p<0.001).

Laboratory data for diabetes and control groups are shown in Table 1. Compared to the NDM individuals, T2DM patients had significantly lower levels of HDL-C, 25(OH)D, free vitamin D, and bioavailable vitamin D and significantly higher levels of glucose, creatinine, ALT, TG, remnant cholesterol, CRP, and HbA1c.

**Table 1:** Biochemical variables of NDM individuals and T2DM patients. *p<0.001, **p<0.05. DM: diabetic; NDM: non-diabetic; T2DM: type 2 diabetes mellitus; ALT: alanine aminotransferase; HDL-C: high-density lipoprotein cholesterol; LDL-C: low-density lipoprotein cholesterol; CRP: C-reactive protein; VDBP: vitamin D binding protein.

	NDM (n = 208)			DM (n = 198)			p-value
	Mean	SD	Minimum-maximum	Mean	SD	Minimum-maximum	
Glucose (mg/dL)	93.4	10	68-127	159	66	45-429	<0.001*
Creatinine (mg/dL)	0.76	0.14	0.47-1.2	0.8	0.16	0.44-1.4	0.002**
ALT (U/L)	21	11.9	6-76	24	12.1	5-64	0.002**
Triglycerides (mg/dL)	127	62	30-422	175	112	42-981	<0.001*
Total Cholesterol (mg/dL)	212	39	85-311	216	44	106-418	0.833
HDL-C (mg/dL)	55	11.8	30-98	49	11.8	21-86	<0.001*
LDL-C (mg/dL)	132	34	19-225	134	37	42-291	0.885
Non-HDL-C (mmol/L)	157	37	25-261	166	42	71-354	0.125
Remnant cholesterol (mg/dL)	25	12	6-69	32	15	8-76	<0.001*
CRP (mg/L)	4	3.9	0.02-26.7	7.3	17.1	0.05-205.8	<0.001*
HbA1c (%)	5.5	0.4	4.5-6.7	7.4	1.6	5.2-13.3	<0.001*
Albumin (g/L)	4.4	0.28	3.7-5.1	4.4	0.27	3.2-5	0.488
VDBP (ng/mL)	136	53	49-420	145	61	49-514	0.346
25(OH)D (ng/mL)	20.6	12	2.1-82.3	19.4	15	1.3-96.7	0.003**
Free vitamin D (pg/mL)	11.4	8.4	0.9-48	10.2	9.5	0.7-71.3	0.005**
Bioavailable vitamin D (ng/mL)	4.5	3.3	0.38-18.66	4	3.6	0.27-26.83	0.005**

Sex-based comparison of remnant cholesterol levels in the entire study group showed that remnant cholesterol levels were significantly higher in men than in women (31±16 vs 27±14 mg/dL, respectively, p=0.024). While this difference was not observed in the NDM and DM groups (p=0.081 and p=0.745, respectively).

The data obtained in separate correlation analysis of the NDM and DM groups are presented in Table 2. In non-diabetic individuals, remnant cholesterol was negatively correlated with 25(OH)D, free vitamin D, and bioavailable vitamin D, but positively correlated with CRP and VDBP. These correlations were not observed in DM patients. However, negative correlations between CRP and free and bioavailable vitamin D and a positive correlation between CRP and VDBP were emerged among DM patients.

**Table 2:** Correlation analysis in NDM individuals and T2DM patients. *p<0.001, **p<0.05. DM: diabetic; NDM: non-diabetic; CRP: C-reactive protein; VDBP: vitamin D binding protein; T2DM: type 2 diabetes mellitus.

NDM individuals		CRP	VDBP	25(OH)D	Free vitamin D	Bioavailable vitamin D
Remnant cholesterol	r	0.277	0.208	-0.146	-0.210	-0.208
	p	<0.001*	0.003**	0.035**	0.002**	0.003**
CRP	r		0.095	-0.083	-0.094	-0.110
	p		0.170	0.235	0.176	0.112
VDBP	r			-0.045	-0.447	-0.453
	p			0.520	<0.001*	<0.001*
DM patients		CRP	VDBP	25(OHD)	Free vitamin D	Bioavailable vitamin D
Remnant cholesterol	r	0.054	0.048	-0.133	-0.113	-0.100
	p	0.452	0.505	0.061	0.112	0.160
CRP	r		0.158	-0.129	-0.205	-0.219
	p		0.028**	0.073	0.004**	0.002**
VDBP	r			-0.032	-0.427	-0.453
	p			0.659	<0.001*	<0.001*

The comparison of independent variables according to the identified remnant cholesterol threshold value (26.56 mg/dL) is presented in Table 3. Within the NDM individuals, subjects with high remnant cholesterol had significantly lower levels of free vitamin D and bioavailable vitamin D compared to those with normal remnant cholesterol (p<0.05). However, the difference in 25(OH)D level was not significant. This relationship was not present among patients in the T2DM group.

**Table 3:** Laboratory analysis stratified by remnant cholesterol cut-off value in NDM individuals and DM patients. *p<0.001, **p<0.05. DM: diabetic; NDM: non-diabetic; T2DM: type 2 diabetes mellitus; CRP: C-reactive protein; VDBP: vitamin D binding protein.

	NDM individuals					DM patients				
	Remnant cholesterol normal (n = 141)		Remnant cholesterol high (n = 67)		p-value	Remnant cholesterol normal (n = 141)		Remnant cholesterol high (n = 67)		p-value
	Median	Minimum-maximum	Median	Minimum-maximum		Median	Minimum-Maximum	Median	Minimum-Maximum	
Age	42	20-69	48	19-67	0.085	55	21-66	55	32-66	0.793
Triglycerides (mg/dL)	94	30-422	186	133-346	<0.001*	103	42-402	197	133-981	<0.001*
CRP (mg/L)	1.6	0.02-26.6	4.1	0.2-26.7	<0.001*	4.2	0.05-205.8	3.6	0.13-56	0.969
Albumin (g/L)	44	37-51	44	37-51	0.441	44	38-51	44	32-50	0.141
VDBP (ng/mL)	121	49-420	145	71-367	0.001**	130	52-514	137	49-299	0.587
Vitamin D (ng/mL)	19.2	2.14-82.28	18.6	3.1-60.76	0.454	16.86	1.32-96.71	13.95	2.55-83.49	0.094
Free vitamin D (pg/mL)	11.3	0.9-48	8.3	1.5-41	0.031**	8.6	0.7-71.3	7.6	1.1-44.8	0.154
Bioavailable vitamin D (ng/mL)	4.61	0.38-18.66	3	0.61-16.82	0.031**	3.26	0.27-26.83	2.93	0.33-18.07	0.199

The results of separate chi-square analyses of the NDM individuals and DM patients to explain the correlation between bioavailable vitamin D and remnant cholesterol are presented in Table 4. Threshold values of 26.56 mg/dL for remnant cholesterol and 3.56 ng/mL for bioavailable vitamin D were used.

**Table 4:** Cross-table between bioavailable vitamin D and remnant cholesterol categories according to the cut-off values. *p<0.05.

NDM individuals		Remnant cholesterol		p-value
		Low (n; %)	High (n; %)	0.014*
Bioavailable vitamin D	Low	57; 58.8	40; 41.2	
	High	84; 75.7	27; 24.3	
DM Patients		Remnant cholesterol		p-value
		Low (n; %)	High (n; %)	0.591
Bioavailable vitamin D	Low	49; 43.4	64; 56.6	
	High	41; 48.2	44; 51.8	

In the NDM group, 41.2% of the individuals with low bioavailable vitamin D level and 24.3% of the individuals with high bioavailable vitamin D level had remnant cholesterol levels above the threshold value (p=0.014). In the DM group, there was no significant correlation between bioavailable vitamin D and remnant cholesterol levels.

The logistic regression analyses used to explain the correlation between bioavailable vitamin D and remnant cholesterol are presented in Table 5. In the control group, those with low levels of bioavailable vitamin D had a 2.01-fold higher risk of high (above the threshold) remnant cholesterol compared to those with normal levels of bioavailable vitamin D (p=0.029), while high CRP levels were associated with a 1.1-fold higher risk of high remnant cholesterol level (p=0.004). In the T2DM group, none of the variables were associated with a significantly higher risk of remnant cholesterol levels above the threshold value.

Subjects with low bioavailable vitamin D level had a 1.69-fold higher risk of high remnant cholesterol compared to those with normal levels of bioavailable vitamin D (p=0.013). Risk of high remnant cholesterol was 2.22 times higher in the presence of T2DM (p<0.001). Evaluation of the interaction between bioavailable vitamin D and T2DM showed that these two factors independently increased the risk of high remnant cholesterol by 2.18- and 2.92-fold, respectively.

**Table 5:** Logistic regression analysis of high remnant cholesterol. Dependent variable is the remnant cholesterol in all models. Model 1: age, gender, CRP, and bioavailable vitamin D were used as independent variables in the controls.  Model 2: age, gender, CRP, and bioavailable vitamin D were used as independent variables in T2DM patients. Model 3: age, gender, CRP, bioavailable vitamin D and T2DM were used as independent variables for the entire study group. *p<0.05. CI: confidence interval; CRP: C-reactive protein; T2DM: type 2 diabetes mellitus.

Independent variables	Odds ratio (95%CI)	p-value
Model 1		
Gender	1.16 (0.57-2.40)	0.677
Age	1.02 (0.10-1.05)	0.098
CRP	1.10 (1.03-1.17)	0.004*
Bioavailable vitamin D	2.01 (1.08-3.76)	0.029*
Model 2		
Gender	0.70 (0.39-1.27)	0.247
Age	1.02 (0.98-1.05)	0.316
CRP	0.97 (0.93-1.01)	0.117
Bioavailable vitamin D	1.35 (0.75-2.43)	0.315
Model 3		
Gender	0.85 (0.54-1.34)	0.412
Age	1.02 (0.10-1.04)	0.070
CRP	0.99 (0.97-1.01)	0.389
Bioavailable vitamin D	2.18 (1.20-3.97)	0.010*
Presence of T2DM	2.92 (1.53-5.58)	0.001*
Bioavailable vitamin D X T2DM	0.60 (0.26-1.39)	0.235

Logistic regression analysis to determine whether insufficient and low levels of total 25(OH)D were associated with remnant cholesterol revealed no significant risk (OR: 1.54, 95%CI: 0.88-2.70, p=0.130).

## Discussion

Diabetic patients exhibit TG metabolism disorders and an atherogenic lipid profile in addition to carbohydrate metabolism disorder [1]. The dyslipidemia accompanying diabetes is characterized by high TG levels and low HDL-C levels [9]. The diabetic patients in the present study had higher TG and remnant cholesterol levels and lower HDL-C levels than the non-diabetic group. The lack of difference in LDL-C levels was supportive of the literature.

Even with statins lowered cardiovascular disease (CVD) risk due to their cholesterol-lowering effect, it has been shown that glycemic control was ineffective in reducing the risk of CVD in diabetic patients [9,10]. TG-rich lipoproteins are thought to be a modifiable risk factor for CVD and are therefore potential therapeutic targets. In randomized controlled studies, it was observed that a TG-lowering effect prevented the development of CVD [11]. The main carrier of circulating TG is VLDL. VLDL metabolism via lipoprotein lipase results in the formation of cholesterol-rich IDL and LDL, the main atherogenic lipoprotein. The cholesterol components of VLDL and IDL are called remnant cholesterol because they are formed as metabolic by-products. Chylomicrons and chylomicron remnants are added postprandially. Some of these atherogenic lipoproteins, namely chylomicrons and VLDL particles greater than 75 nm in diameter, remain in the vessels, while small VLDL, IDL, and chylomicron remnants may pass into the arterial intima by simple concentration-dependent filtration. However, they cannot penetrate the elastic lamina of the arterial media, become trapped in the intima and taken up directly by macrophages [12,13]. Macrophage infiltration of the vessel wall promotes the formation of foam cells. Furthermore, the increase of remnant lipoproteins in the arterial wall is related to low degree inflammation and may contribute to atherogenesis [3]. Recently studies in diabetic patients have shown that, remnant cholesterol is the most important independent risk factor of CAD and independently associated with in-stent restenosis [14]. It has also been shown to be an independent risk factor for CAD and DM in menopausal women [3].

Nowadays, more importance is placed on TG-rich lipoproteins because we are satiated most of the day and these lipoproteins are dominant postprandially. Using direct measurement it was found that one third of nonfasting plasma cholesterol was present in remnant lipoproteins [13]. It has been shown that, assessment of such lipoproteins at postprandial state may predict future CVD events, on the other hand, assessment at fasting state is important for accurate diagnosis [15]. Nagata et al [16], found that fasting levels of TG-rich lipoproteins may be an indicator of postprandial hyperlipidemia.

Directly measured remnant cholesterol without fractioning and calculated remnant cholesterol were found to be very well correlated [3]. Nakajima et al [17], classified fasting remnants according to their content as remnant lipoprotein TG and remnant lipoprotein cholesterol, and used direct measurement to quantify the two different analytes. For values below 400 mg/dL in the control group, they identified 95th percentile threshold values of 20 mg/dL for remnant lipoprotein TG and 7.5 mg/dL for remnant lipoprotein cholesterol. In the VLDL4 study, remnant cholesterol threshold value calculated by isolating VLDL and IDL fractions was 32 mg/dL at the 75th percentile and 41 mg/dL at the 90th percentile [18]. These differences may be a result of the fractioning methods used in the analysis phase. In the non-diabetic group in our study, the remnant cholesterol threshold value was identified as 26.56 mg/dL for individuals with TG below 150 mg/dL and total cholesterol below 200 mg/dL as suggested by NCEP. In diabetic patients, the remnant cholesterol cut-off value for in-stent restenosis was shown to be 19.4 mg/dL using the formula we used [14].

A relationship between vitamin D deficiency and insulin resistance has been demonstrated in meta-analyses [4]. Evidence that may explain the underlying mechanism is decreased glucose consumption in the skeletal muscle and the presence of vitamin D receptor and 1-alpha hydroxylase activity in the pancreas of diabetes patients [19,20]. Furthermore, the results of a meta-analysis indicated that sufficient 25(OH)D levels reduced the risk of developing T2DM [4]. Similarly, the diabetic patients in our study had lower total vitamin D levels than the non-diabetic participants.

Several theories have been suggested in response to observations that vitamin D supplementation did not improve atherogenic lipid profile or insulin resistance [4]. Among these are the recent hypotheses that measured 25(OH)D concentrations do not reflect actual activity and that bioavailable vitamin D levels should be considered instead [21]. The majority of circulating vitamin D is bound by VDBP. This complex is transported in the circulation by VLDL, and vitamin D levels are therefore closely related to TG metabolism [22]. For this reason, the current study analyzed the association between bioavailable vitamin D and remnant cholesterol in patients with T2DM, whose TG metabolism is altered.

In a recent study, low bioavailable and free vitamin D have been shown to be significantly associated with cardiovascular mortality [23]. In contrast, serum total 25(OH)D level was not associated. Pelczyńska et al [7], found that patients with metabolic syndrome had lower levels of 25(OH)D, free and bioavailable vitamin D, and similar levels of VDBP compared to patients without metabolic syndrome. In our study, the presence of T2DM was not associated with differences in VDBP concentrations, but diabetic patients had lower levels of 25(OH)D, free and bioavailable vitamin D compared to non-diabetics. An earlier study conducted in Turkey reported no differences of VDBP levels between DM and control groups, comparable to our results [24].

The albumin-bound and free forms of vitamin D are bioavailable and responsible for its activity. According to the free hormone hypothesis, this bioavailable vitamin D affects metabolism, rather than total 25(OH)D [21]. In addition to the free hormone hypothesis, it has been argued that personal metabolic profile, hormonal balance differences, vitamin D sequestration in adipose tissue, and genetic polymorphisms may alter vitamin D activity. It has also been suggested that there may be an inverse relationship between lipid profile and vitamin D, with lipid profile affecting vitamin D [25-28].

Epidemiological studies and meta-analyses have unequivocally demonstrated the existence of an association between diabetes, lipid profile, and vitamin D, but causality has yet to be established [27]. In our study, remnant cholesterol was negatively correlated with 25(OH)D, free vitamin D, and bioavailable vitamin D in non-diabetic individuals. However, these inverse relationships were not observed in patients with T2DM. This may be related to the inflammation that occurs in diabetes, because we observed positive correlations between VDBP and CRP levels in the T2DM group. Furthermore, in our study, T2DM patients exhibited higher CRP levels than the non-diabetic participants. These correlations appeared among diabetic patients suggests that bioavailable vitamin D may be reduced in diabetes due to the inflammation process.

Epidemiological studies have also revealed a link between reduced vitamin D and lipid profile, remnant cholesterol, and subsequent development of CVD [1,4]. Remnant cholesterol promotes atherosclerosis formation through several mechanisms, including upregulation of endothelial proinflammatory cytokines, monocyte migration and activation, and overproduction of prothrombotic factors [29]. These factors are known to increase VDBP production. If remnant cholesterol does in fact increase VDBP levels and reduce bioavailable vitamin D, then the ineffectiveness of vitamin D supplementation is expected. Our finding that high remnant cholesterol was associated with high VDBP and CRP levels and low bioavailable vitamin D level in non-diabetic people supports the mechanisms discussed above. In diabetic people, underlying inflammation may have disrupted this mechanism.

According to our logistic regression analysis, 25(OH)D was not a significant determinant of remnant cholesterol levels. Low bioavailable vitamin D, however, was associated with a 2.185-fold higher risk of high remnant cholesterol level, independent of the presence of T2DM.

In this study, high CRP levels and bioavailable vitamin D deficiency significantly increased remnant cholesterol levels in the non-diabetic group. Although the mechanism of this relation has not yet been determined in humans, preclinical studies showed that vitamin D enhances lipid catabolism by way of nuclear receptors, PPAR and LXR [25,26]. VDBP polymorphism and use of vitamin D supplementation may be among the reasons that this relation applies to bioavailable vitamin D but not 25(OH)D. We believe that the correlation between bioavailable vitamin D and remnant cholesterol may be disrupted in diabetic patients due to diabetes-related inflammation.

These findings support the association between remnant cholesterol and bioavailable vitamin D, although the nature of the relationship is not clear. The culprit mechanism will be elucidated with the completion of ongoing drug studies aimed at reducing TG and elevating vitamin D levels. In both cases, we believe that VDBP measurement and bioavailable vitamin D estimation will be helpful for determining actual vitamin D status.

The main limitation of this study was the cross-sectional structure. We excluded participants receiving lipid-lowering therapy, but we did not exclude vitamin D supplementation. Another limitation was the lack of clinical data such as menopausal status. Future studies may evaluate the factors identified in our study in order to determine causality in the relationship we observed independent of diabetes. Finally, in our study, we used the monoclonal antibody VDBP test, which was reported to be affected by the genotype [30]. We calculated free and bioavailable vitamin D concentrations using the Bikle method. The free vitamin D calculated from this equation was demonstrated in close agreement with the directly measured value [21].

The strength of our study was that the participants were from three major medical centers in the city of Izmir. There are numerous studies demonstrating an association between vitamin D and lipid profile; however, our literature search yielded no publications about the correlation between bioavailable vitamin D and remnant cholesterol levels.

The results of this study suggest the benefit of using remnant cholesterol and extended lipid panel in addition to the standard lipid profile when evaluating patients for cardiovascular risk, restricting intake of drugs and food that elevate TG levels in patients at risk, and optimizing TG levels in diabetic patients.

## Conclusions

Bioavailable vitamin D level rather than total 25(OH)D level was associated with high remnant cholesterol, independent of the presence of T2DM. Randomized controlled trials are needed to explain the causal association between bioavailable vitamin D and remnant cholesterol. We believe that the inflammation observed in Diabetes Mellitus may increase the concentrations of VDBP and a decrease in bioavailable vitamin D levels. Measurement of vitamin D binding protein provides additional information about the actual vitamin D status. Also expanded lipid panel analysis, including remnant lipoprotein cholesterol, etc., can be useful in routine clinical practice.
